# Hemodynamic effects of extended prone position sessions in ARDS

**DOI:** 10.1186/s13613-018-0464-9

**Published:** 2018-12-07

**Authors:** Martin Ruste, Laurent Bitker, Hodane Yonis, Zakaria Riad, Aurore Louf-Durier, Floriane Lissonde, Sophie Perinel-Ragey, Claude Guerin, Jean-Christophe Richard

**Affiliations:** 1 Service de Réanimation Médicale, Hôpital De La Croix Rousse, Hospices Civils de Lyon, 103 Grande Rue de la Croix Rousse, 69004 Lyon, France; 2 Université de Lyon, Université LYON I, Lyon, France; 3 IMRB, INSERM 955Eq13, Créteil, France; 4 CREATIS INSERM 1044 CNRS 5220, Villeurbanne, France

**Keywords:** Acute respiratory distress syndrome, Cardiac output, Prone position, Positive end-expiratory pressure, Transpulmonary thermodilution, Cardiac preload

## Abstract

**Background:**

Hemodynamic response to prone position (PP) has never been studied in a large series of patients with acute respiratory distress syndrome (ARDS). The primary aim of this study was to estimate the rate of PP sessions associated with cardiac index improvement. Secondary objective was to describe hemodynamic response to PP and during the shift from PP to supine position.

**Methods:**

The study was a single-center retrospective observational study, performed on ARDS patients, undergoing at least one PP session under monitoring by transpulmonary thermodilution. PP sessions performed more than 10 days after ARDS onset, or with any missing cardiac index measurements before (T_1_), at the end (T_3_), and after the PP session (T_4_) were excluded. Changes in hemodynamic parameters during PP were tested after statistical adjustment for volume of fluid challenges, vasopressor and dobutamine dose at each time point to take into account therapeutic changes during PP sessions.

**Results:**

In total, 107 patients fulfilled the inclusion criteria, totalizing 197 PP sessions. Changes in cardiac index between T_1_ and T_2_ (early response to PP) and between T_1_ and T_3_ (late response to PP) were significantly correlated (*R*^2^ = 0.42, *p* < 0.001) with a concordance rate amounting to 85%. Cardiac index increased significantly between T_1_ and T_3_ in 49 sessions (25% [95% confidence interval (CI_95%_) 18–32%]), decreased significantly in 46 (23% [CI_95%_ 16–31%]), and remained stable in 102 (52% [CI_95%_ 45–59%]). Global end-diastolic volume index (GEDVI) increased slightly but significantly from 719 ± 193 mL m^−2^ at T_1_ to 757 ± 209 mL m^−2^ at T_3_ and returned to baseline values at T_4_. Cardiac index and oxygen delivery decreased slightly but significantly from T_3_ to T_4_, without detectable increase in lactate level. Patients who increased their cardiac index during PP had significantly lower CI, GEDVI, global ejection fraction at T_1_, and received significantly more fluids than patients who did not.

**Conclusion:**

PP is associated with an increase in cardiac index in 18% to 32% of all PP sessions and a sustained increase in GEDVI reversible after return to supine position. Return from prone to supine position is associated with a slight hemodynamic impairment.

**Electronic supplementary material:**

The online version of this article (10.1186/s13613-018-0464-9) contains supplementary material, which is available to authorized users.

## Background

Prone position (PP) sessions of at least 16 h are now an established treatment in acute respiratory distress syndrome (ARDS) patients with PaO_2_/FIO_2_ ratio below 150 mmHg, with a clear beneficial effect on mortality [1, 2]. However, PP impact on hemodynamics has only been ascertained in small studies [3–11], most of which have been performed before the era of protective ventilation and with shorter PP sessions.

While virtually all these studies failed to identify any impact of PP on cardiac index (CI), two recent studies [12, 13] have identified a positive hemodynamic effect of PP in two clinical scenarios. First, PP may improve CI in patients presenting with acute cor pulmonale (ACP), in relation to the unloading of the right ventricle [12]. Second, PP may also improve venous return and subsequently cardiac preload within 20 min after postural change, hence increasing CI in patients presenting preload responsiveness [13]. However, the persistence of this effect during prolonged PP sessions remains to date unknown. Since prevalence of ACP has been ascertained to 22% in a large series of 752 ARDS patients [14] and preload responsiveness before PP was identified in 50% of the patients [13], CI should increase with PP in a substantial fraction of ARDS patients, in conflict with previous reports.

Furthermore, if the shift from supine position (SP) to PP indeed increases CI by increasing venous return, we hypothesize that the shift from PP to SP may have an opposite effect, which remains unreported by previous studies [3, 4, 6, 7, 9, 11], although strongly underpowered to detect such an effect.

To our knowledge, hemodynamic response to prolonged PP sessions has never been studied in a large series of ARDS patients.

## Methods

### Study aim

The primary aim of the study was to estimate the rate of PP sessions associated with an improvement in CI. Secondary objective was to describe hemodynamic response before, during, and after a PP session.

### Study design

This single-center retrospective observational study reports data from patients hospitalized between July 2012 and December 2016 in an academic medical intensive care unit (ICU). The study protocol was approved by an Ethics Committee (CPP Sud-Est II, IRB 9118), which waived the requirement for informed consent.

### Patients

To be eligible, the subjects had to fulfill all the following inclusion criteria: ARDS according to the Berlin definition [15], application of at least one PP session, and hemodynamic monitoring by the PiCCO^®^ device (Pulsion Medical Systems, Feldkirchen, Germany). Non-inclusion criteria were the following: age < 18 years, advanced directives to withhold or withdraw life-sustaining treatment initiated before PP session, and previous inclusion during prior ICU admission. PP sessions performed more than 10 days after ARDS onset, or performed during extracorporeal membrane oxygenation, or during which decision to withhold or withdraw life-sustaining treatment was taken, or with any missing CI measurements before, at the end, or after the PP session were excluded. Multiple PP sessions per patient during the same ICU stay could be analyzed should the eligibility criteria be fulfilled during sessions.

### Protocol description

Since 2011, ARDS management is routinely performed in our ICU according to the protocol used in the PROSEVA study [1] as follows: protective ventilation with a tidal volume of 6 mL kg^−1^ predicted body weight, positive end-expiratory pressure (PEEP) setting using a PEEP–FiO_2_ table [16], administration of neuromuscular blocking agent during 48 h if PaO_2_/FiO_2_ < 150 mmHg [17], and daily PP during at least 16 h until achievement of a PaO_2_/FiO_2_ ≥ 150 mmHg with a PEEP ≤ 10 cm H_2_O and a FiO_2_ ≤ 60% in the SP. Hemodynamic monitoring, using the PiCCO^®^ device, is routinely used whenever severe shock is associated with ARDS [18].

PiCCO^®^ monitoring was performed using a femoral arterial catheter, connected to an Intellivue MP40 monitor (Philips Healthcare, Andover, MA, USA) equipped with the PiCCO^®^ module. PiCCO^®^ calibrations were performed in SP or PP with a horizontal bed position at least every 4 h, with a triplicate intravenous injection of 15 mL cold 9‰ sodium chloride [19, 20] through a venous catheter in the superior vena cava territory.

T_1_, T_2_, T_3,_ and T_4_ were, respectively, defined as the times of PiCCO^®^ calibration performed in the SP closest to PP onset, during PP closest to session onset, during PP closest to session ending, and after PP.

### Data collection

The following variables were recorded at ICU admission or ARDS onset: demographic data, SAPS II score [21], ARDS severity [15], and risk factors. Occurrence of ACP on echocardiography was recorded at ICU admission and during follow-up in all patients and defined by the association of septal dyskinesia and right ventricle dilation (surface ratio of right ventricle over left ventricle greater than 0.6) [14] at any time. The following variables were recorded on the day of each PP session: SOFA score [22], time from ICU admission to PP session, cumulative fluid balance at PP session onset and during PP session, and ARDS adjunctive therapies. Hemodynamic variables were recorded at T_1_, T_2_, T_3,_ and T_4_ of each PP session. Respiratory variables were recorded at T_1_, T_3_, and T_4_ of each PP session. Missing data per variable are reported in Additional file 1: Table S1.

### Data analysis

Significant changes in CI and global end-diastolic volume index (GEDVI) were deemed present for variations greater than ± 15% [19]. Patients with an increase in PaO_2_/FIO_2_ ≥ 20 mmHg or a decrease in PaCO_2_ ≥ 1 mmHg at T_3_ relative to T_1_ were classified as O_2_ or CO_2_ responders to PP, respectively [23]. Patients with both increase in PaO_2_/FiO_2_ ≥ 20 mmHg and decrease in PaCO_2_ ≥ 1 mmHg at T_3_ relative to T_1_ were classified as O_2_ and CO_2_ responders to PP. Oxygen delivery was computed as previously described [24].

### Statistical analysis

Statistical analysis was performed using R software with packages Lme4 [25], Lmertest [26], multcomp [27], MultinomialCI [28], and OptimalCutpoints [29].

We defined the PP session as the statistical unit. Power of the study was computed using the normal approximation confidence interval method. We calculated that with a sample size of at least 196 PP sessions, the study would provide at worst a ± 7% precision in the 95% confidence interval (CI_95%_) of the rate of PP sessions associated with CI improvement.

Numerical variables are expressed as mean ± standard deviation and categorical variables as counts with corresponding percentages. CI_95%_ for multinomial proportions was computed using Sison and Glaz method [30]. Linear mixed models were used to take into account both measurement repetition during a PP session and multiple PP sessions per patient. Changes in hemodynamic variables over time were tested after adjustment for volume of fluid challenges, vasopressor and dobutamine doses to take into account therapeutic changes between time points. Multiple comparisons between groups were performed using Holm method. Diagnostic performance was assessed by computation of area under ROC curve (AUC) [31]. CI_95%_ for AUC was computed using the Delong method. The optimal cutoff points were computed by maximizing the Youden index. A *p* value below 0.05 was chosen for statistical significance and computed using parametric bootstrapping [32].

## Results

### Population

**Fig. 1:**
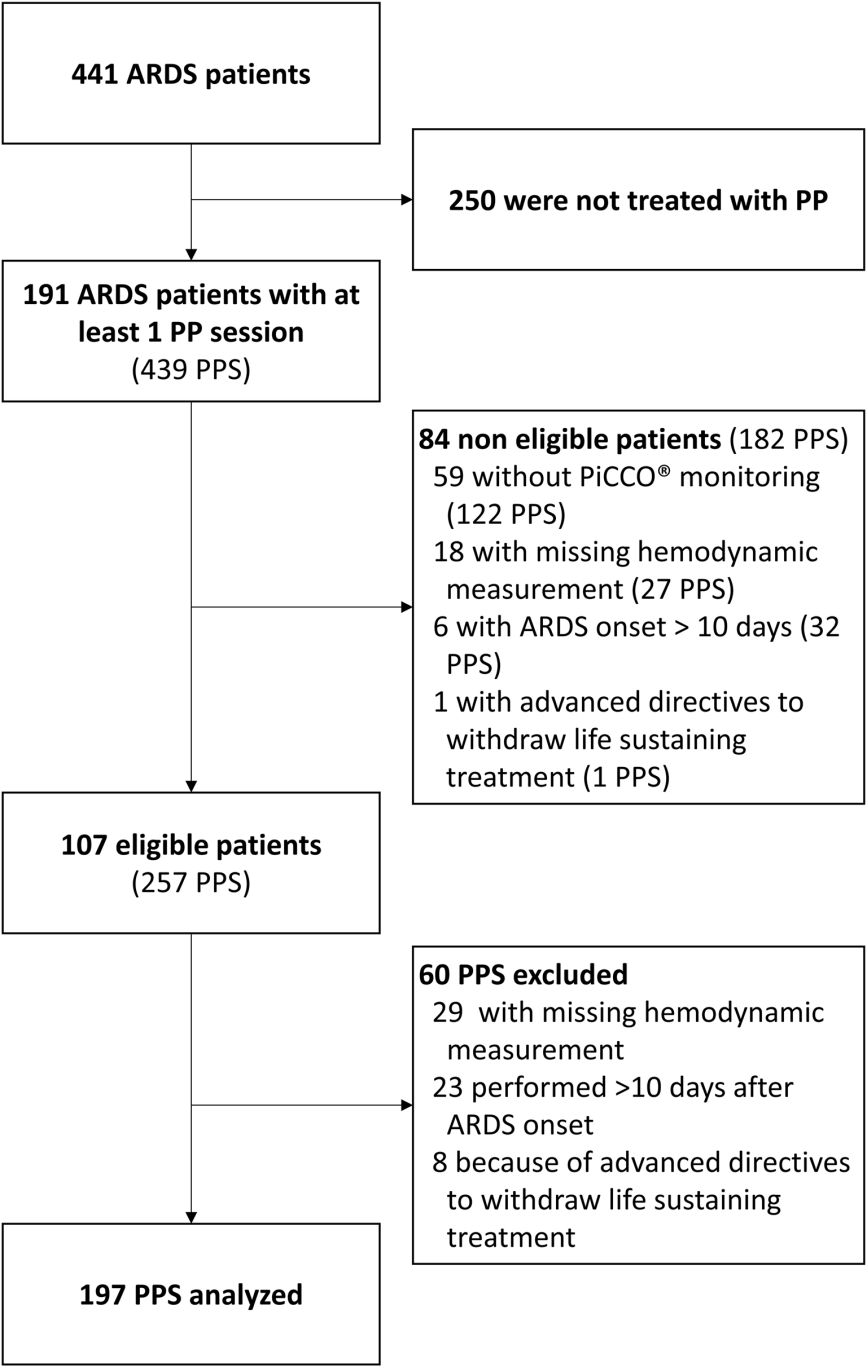
Study flowchart. *ARDS* acute respiratory distress syndrome, *PPS* prone positioning session

A total of 191 patients fulfilled the inclusion criteria over the study period, among which 84 presented with non-inclusion criteria (Fig. 1). Characteristics of the 107 patients included in the study are reported in Table 1. Ten patients (9%) presented with ACP during follow-up, and all patients had at least one ultrasound evaluation before the first PP session of the study, with a delay amounting to 1 ± 2 days. In total, 60 PP sessions were excluded, and 197 were hence considered for analysis, whose detailed characteristics are reported in Table 2.

**Table 1:** Patients’ characteristics

Variable	Patients characteristics *n* = 107
Age (year)	65 ± 12 (35; 89)
Male sex	73 (68%)
Body mass index (kg m^−2^)	29 ± 7 (16; 54)
SAPS II	62 ± 18 (32; 118)
Admission category	
Medical	103 (96%)
Emergent surgery	2 (2%)
Elective surgery	1 (1%)
Trauma	1 (1%)
ARDS severity	
Moderate	30 (28%)
Severe	77 (72%)
Time between ICU admission and ARDS onset	2 ± 4 (− 3; 27)
ARDS risk factors^a^	
Pneumonia	79 (74%)
Aspiration	34 (32%)
Extra pulmonary sepsis	10 (9%)
None	3 (3%)
Acute cor pulmonale	10 (9%)

Values are count (percentage) or mean ± standard deviation (extreme values)

*ARDS* acute respiratory distress syndrome, *ICU* intensive care unit, *SAPS II* simplified acute physiology score II, *SOFA* sepsis-related organ failure assessment

^a^Total > 100% since multiple risk factors could be identified per patient

**Table 2:** Characteristics of prone positioning sessions

Variables	Session characteristics *n* = 197
Number of PP sessions per patient	2 ± 2
Time between ARDS onset and PP session onset (day)	3 ± 3
Duration of PP session (h)	16 ± 3
SOFA score	15 ± 4
Body weight at PP session onset (kg)	86 ± 19
Cumulative fluid balance at PP session onset (kg)	2.3 ± 6.5
Fluid balance during PP session (kg)	0.8 ± 3.3
Fluid challenge during PP session	78 (40%)
Volume of fluid challenges during PP session (mL)	505 ± 1069
Renal replacement therapy	84 (43%)
Dobutamine administration	62 (31%)
Vasopressor administration	166 (84%)
Inhaled nitric oxide	39 (20%)
Neuromuscular blocking agents	182 (92%)
O_2_ responders to PP	157 (80%)
CO_2_ responders to PP	97 (49%)
O_2_ and CO_2_ responders to PP	85 (43%)

Values are count (percentage) or mean ± standard deviation

CO_2_ responders to PP = patients in which PaCO_2_ decreases by at least 1 mmHg between end of PP session and before PP session. O_2_ responders to PP = patients in which PaO_2_/FiO_2_ increases by at least 20 mmHg between end of PP session and before PP session. O_2_ and CO_2_ responders to PP = patients in which PaO_2_/FiO_2_ increases by at least 20 mmHg and PaCO_2_ decreases by at least 1 mmHg between end of PP session and before PP session

*PP* prone position, *SOFA* sepsis-related organ failure assessment

### Hemodynamic and respiratory measurements

Actual T_1_ measurements were recorded 2 ± 2 h before PP onset, T_2_ at 3 ± 2 h after PP onset, T_3_ at 13 ± 3 h after PP onset, and T_4_ at 2 ± 2 h after return to SP.

Hemodynamic and respiratory measurements are reported in Tables 3 and 4, respectively. CI was not significantly modified during PP at both T_2_ and T_3_ after adjustment for fluid challenges, vasopressor and dobutamine dose, but significantly decreased after return to SP. Adjusted GEDVI increased slightly but significantly during PP and returned to baseline values at T_4_. Adjusted oxygen delivery decreased slightly but significantly after return to SP, without significant increase in lactate level. Response to PP was not significantly different between successive PP sessions, as we did not find any significant interaction between time points within session and successive PP sessions for all hemodynamic and respiratory variables.

**Table 3:** Hemodynamic parameters during prone positioning session

Variables	T_1_	T_2_	T_3_	T_4_
HR (min^−1^)	98 ± 23	99 ± 20^a^	96 ± 20	95 ± 21
MAP (mmHg)	76 ± 10	79 ± 12^c^	77 ± 12	78 ± 14
CVP (cm H_2_O)	13 ± 5	14 ± 5^c^	13 ± 5	14 ± 5
CI (L min m^−2^)	3.5 ± 1.3^a^	3.4 ± 1.2^a^	3.4 ± 1.1^a^	3.2 ± 1.1
GEF (%)	21 ± 7	20 ± 7^c^	21 ± 8	21 ± 7
EVLWI (mL kg^−1^ PBW)	13.8 ± 4.4	14.2 ± 4.7^a^	13.7 ± 4.7	13.1 ± 4.0
PVPI	2.6 ± 1.0^b^	2.5 ± 0.9	2.4 ± 0.9	2.5 ± 0.9
GEDVI (mL m^−2^)	719 ± 193	738 ± 185^a,c^	757 ± 209^a,c^	714 ± 200
CFI (min^−1^)	5.0 ± 1.9	4.8 ± 1.8^c^	4.8 ± 1.8^c^	4.7 ± 1.8^c^
PTV (mL)	1163 ± 362^a^	1189 ± 359^a^	1173 ± 354^a^	1116 ± 327
ITTV (mL)	2539 ± 653	2602 ± 638^a,c^	2619 ± 664^a,c^	2482 ± 655
DO_2_ (mL min m^−2^)	416 ± 145	NA	414 ± 139	387 ± 126^b,c^
Vasopressor dose* (µg kg min^−1^)	0.92 ± 1.66	0.92 ± 2.04	0.84 ± 1.67^c^	0.88 ± 1.76^c^
Dobutamine dose** (µg kg min^−1^)	2.6 ± 6.0	2.6 ± 5.8	2.8 ± 6.1	3.0 ± 6.2
Volume of fluid challenge since preceding time point*** (mL)	NA	158 ± 519	224 ± 566	123 ± 340

Values are mean ± standard deviation. All statistical tests are performed after adjustment for volume of fluid challenges since preceding time point, vasopressor and dobutamine dose unless specifically stated

CI, cardiac index; CFI, cardiac function index; CVP, central venous pressure; DO_2_, oxygen delivery; EVLWI, extravascular lung water index; GEDVI, global end-diastolic volume index; GEF, global ejection fraction; HR, heart rate; ITTV, intrathoracic thermal volume; MAP, mean arterial pressure; NA, not available; PBW, predicted body weight; PTV, pulmonary thermal volume; PVPI, pulmonary vascular permeability index; T_1_, before prone position, T_2_, beginning of prone position session; T_3_, end of prone position session; T_4_, after prone position session

^a^*p* < 0.05 versus T_4_; ^b^* p* < 0.05 versus T_3_; ^c^ *p* < 0.05 versus T_1_

* Adjustment for volume of fluid challenges since preceding time point and dobutamine dose only; ** adjustment for volume of fluid challenges since preceding time point and vasopressor dose only; *** not tested for statistical significance

**Table 4:** Respiratory parameters during prone positioning session

Variables	T_1_	T_3_	T_4_
VT (mL kg^−1^ PBW)	6.2 ± 0.7	6.2 ± 0.8	6.1 ± 0.8
RR (min^−1^)	29 ± 5	29 ± 5	29 ± 5
I:E ratio (%)	42 ± 11	40 ± 9	39 ± 9
PEEP (cm H_2_O)	10 ± 3^a,b^	9 ± 3	9 ± 3
PEEPtot (cm H_2_O)	11 ± 2	10 ± 3	10 ± 3
Pplat (cm H_2_O)	23 ± 4	22 ± 5^c^	22 ± 4^c^
Δ*p* (cm H_2_O)	12 ± 4	11 ± 4	11 ± 3
pH	7.35 ± 0.10	7.38 ± 0.09^a,c^	7.37 ± 0.09^c^
PaCO_2_ (mmHg)	45 ± 10	43 ± 11	44 ± 10
PaO_2_/FiO_2_	112 ± 28	179 ± 62^a,c^	153 ± 60^c^
Lactate (mmol L^−1^)	3.6 ± 3.2	3.3 ± 3.0	3.2 ± 3.0
Hemoglobin (g L^−1^)	101 ± 22	98 ± 21^c^	98 ± 20^c^

Values are mean ± standard deviation

Δ*p*, driving pressure; FiO_2_, inspired oxygen fraction; I:E ratio, inspiratory-to-expiratory time ratio; PaCO_2_, partial pressure of arterial carbon dioxide; PaO_2_, partial pressure of arterial oxygen; PBW, predicted body weight; PEEP, external PEEP; PEEPtot, total PEEP of the respiratory system; Pplat, plateau pressure of the respiratory system; RR, respiratory rate; T_1_, before prone position, T_3_, end of prone position session; T_4_, after prone position session; VT, tidal volume

^a^*p* < 0.05 versus T_4_; ^b^* p* < 0.05 versus T_3_; ^c^* p* < 0.05 versus T_1_

### Early hemodynamic response to prone position at T_2_

**Fig. 2:**
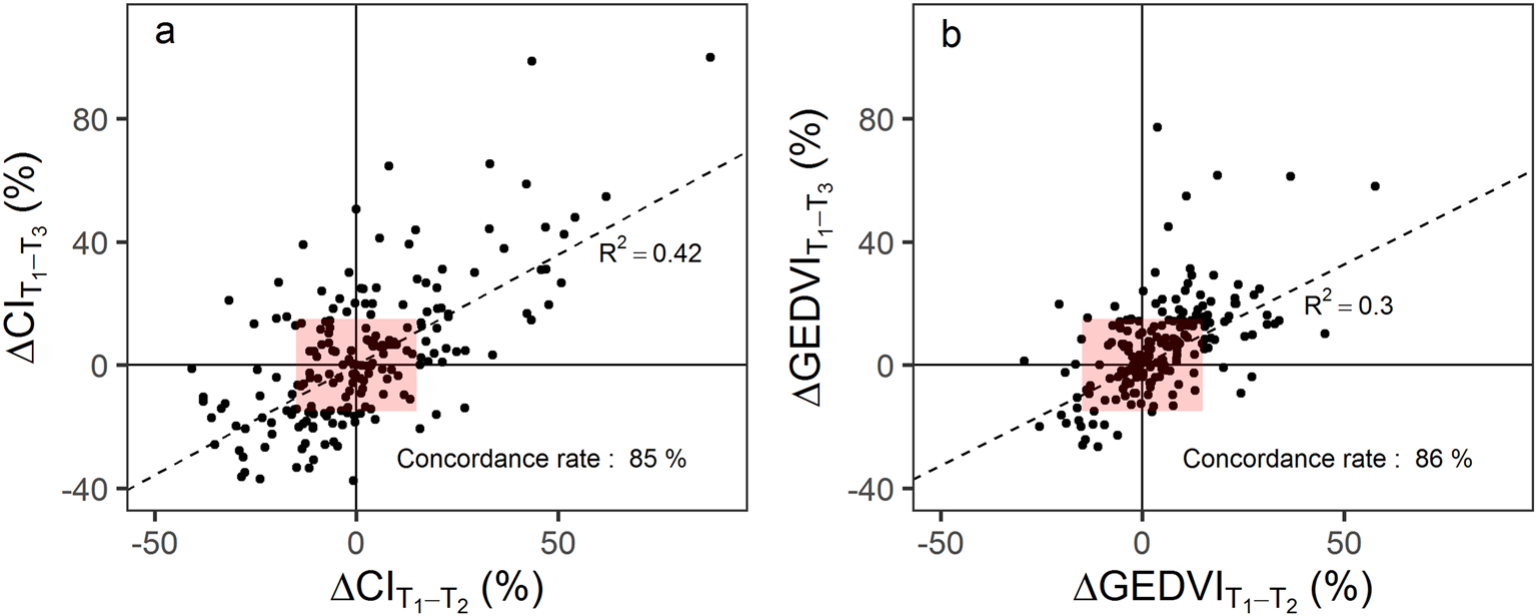
Early versus late changes in CI (**a**) and GEDVI (**b**) during prone position. Symbols are individual values for each prone position session. Broken lines are regression lines performed on the whole dataset with corresponding *R*^2^. Concordance rate is the percentage of data points falling into one of the two quadrants of agreement (i.e., upper right and lower left quadrants in which hemodynamic variations between T_1_ and T_2_ and between T_1_ and T_3_ have the same directional changes). Red rectangles are exclusion zones for computation of concordance rate, excluding data points with changes in both variables below 15%. ΔCI_T1–T2_: change in CI between T_1_ and T_2_; ΔCI_T1–T3_: change in CI between T_1_ and T_3_; ΔGEDVI_T1–T2_ = change in GEDVI between T_1_ and T_2_; ΔGEDVI_T1–T3_ = change in GEDVI between T_1_ and T_3_

Between T_1_ and T_2_, CI increased significantly in 42 sessions (22% [CI_95%_ 15–29%]), decreased significantly in 33 (17% [CI_95%_ 10–24%]), and remained stable in 119 (61% [CI_95%_ 55–68%]). Changes in CI between T_1_ and T_2_ (early response) and between T_1_ and T_3_ (late response) were significantly correlated (*R*^2^ = 0.42, *p* < 0.001, Fig. 2a) with a concordance rate amounting to 85%. Similar results were obtained with changes in GEDVI (Fig. 2b).

### Late hemodynamic response to prone position at T_3_

Between T_1_ and T_3_, CI increased significantly in 49 sessions (25% [CI_95%_ 18–32%]), decreased significantly in 46 (23% [CI_95%_ 16–31%]), and remained stable in 102 (52% [CI_95%_ 45–59%]). Patients who increased their CI during PP had significantly lower CI, GEDVI, global ejection fraction at T_1_, and received significantly more fluids than patients who did not (Additional file 2: Table S2). Patients who decreased their CI between T_1_ and T_3_ had significantly higher CI and GEDVI at T_1_ and received significantly more vasopressors than patients who did not. Fluid balance during PP, oxygenation, and/or carbon dioxide response to PP was not significantly associated with classification of CI response. CI at T_1_ was the variable with the best diagnostic performance to predict CI increase (AUC = 0.79 [CI_95%_ 0.73–0.86]) and decrease (AUC = 0.68 [CI_95%_ 0.59–0.77]) between T_1_ and T_3_ (Additional file 3: Table S3; Additional file 4: Table S4). A CI below 2.8 L min m^−2^ had a sensitivity of 0.69 [CI_95%_ 0.55–0.82] and a specificity of 0.76 [CI_95%_ 0.69–0.83] to predict an increase in CI greater than 15% at T_3_. A CI above 3.5 L min m^−2^ had a sensitivity of 0.63 [CI_95%_ 0.48–0.77] and a specificity of 0.70 [CI_95%_ 0.62–0.77] to predict a decrease in CI greater than 15% at T_3_.

CI response to PP was highly heterogeneous among successive sessions in the 40 patients studied repeatedly (Additional file 5: Figure S1).

Change in CI (ΔCI) and GEDVI (ΔGEDVI) between T_1_ and T_3_ were weakly correlated (*R*^2^ = 0.14, *p* < 0.001, Additional file 6: Figure S2), while correlations were substantially higher in the following subgroups of patients based on their changes in cardiac function index between T_1_ and T_3_ (ΔCFI = ΔCI/ΔGEDVI): patients with ΔCFI ≥ 15% (i.e., with CI increase not fully explained by an increase in GEDVI), patients with − 15% < ΔCFI < 15% (i.e., with CI changes explained by changes in GEDVI or no variation in both GEDVI and CI), and patients with ΔCFI ≤ − 15% (i.e., with CI decrease not fully explained by a decrease in GEDVI).

**Fig. 3:**
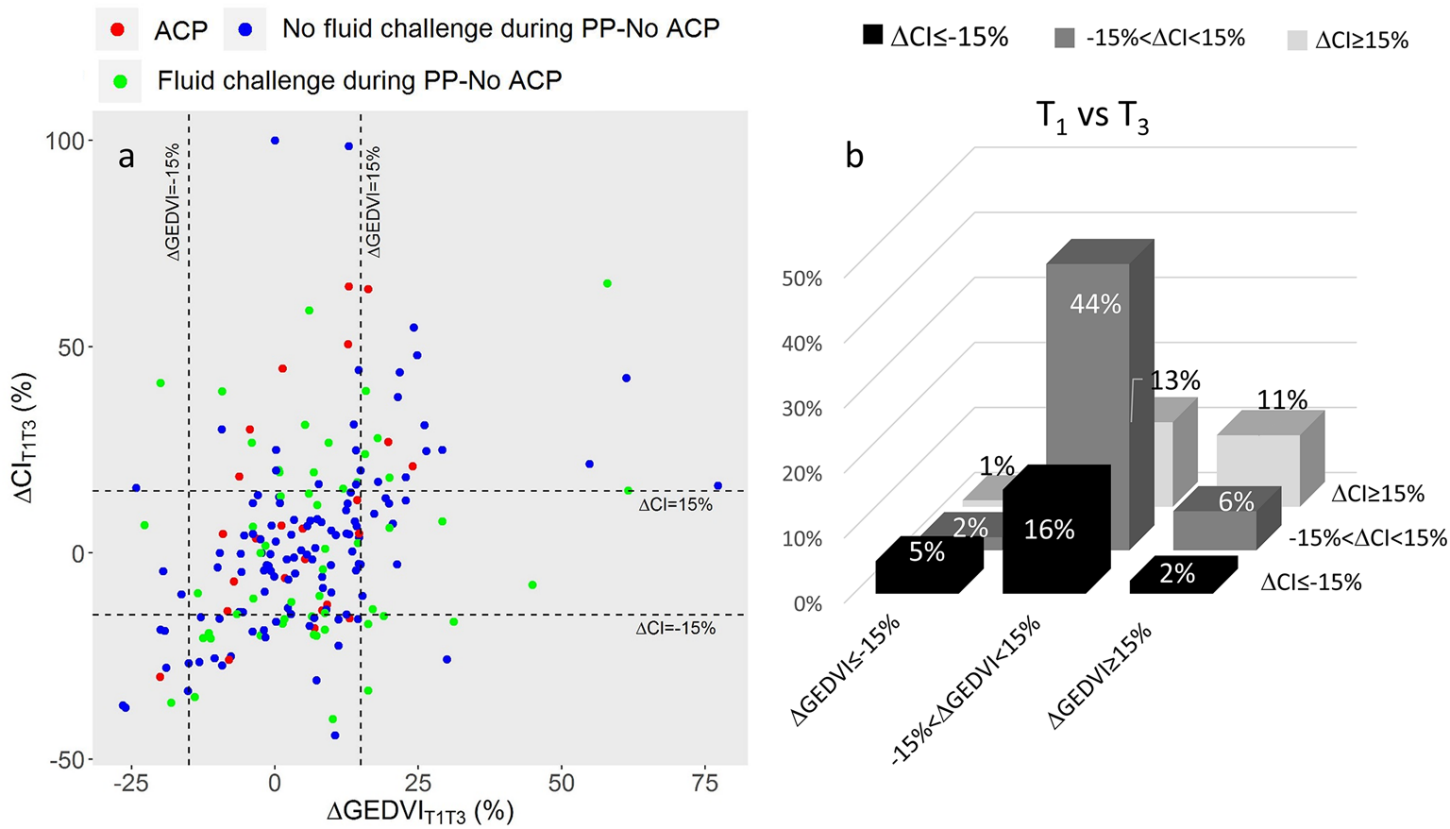
Hemodynamic pattern as a function of ΔCI and ΔGEDVI between T_1_ and T_3_. **a** Symbols are individual values for each prone position session. Broken lines are threshold values for significant changes in CI (+ or − 15%) and GEDVI (+ or − 15%). Red dots refer to patients with ACP. Blue dots refer to patients without ACP and without fluid challenge between T_1_ and T_3_. Green dots refer to patients without ACP and with any fluid challenge between T_1_ and T_3_. **b** Bars are percentage of patients falling in each category. CI, cardiac index; GEDVI, global end-diastolic volume index; ACP, acute cor pulmonale; ΔCI, change in CI; ΔGEDVI, change in GEDVI; PP, prone position; T_1_, before prone position; T_3_, end of prone position session

Changes in CI and GEDVI beyond significant thresholds between T_1_ and T_3_, or T_3_ and T_4_, were used to classify sessions into nine categories (Fig. 3), in an attempt to identify groups with CI variations related or unrelated to changes in GEDVI. CI increase between T_1_ and T_3_ was associated with GEDVI increase in 11% of the sessions (mainly during sessions without concomitant fluid challenge), with stable GEDVI in 13%, and with GEDVI decrease in 1%. Virtually all PP sessions performed on patients with ACP were associated with no change or an increase in CI between T_1_ and T_3_ (Fig. 3). CI decrease between T_1_ and T_3_ was mainly associated with stable GEDVI occurring in 16% of the sessions.

### Hemodynamic response during the shift from prone to supine position

**Fig. 4:**
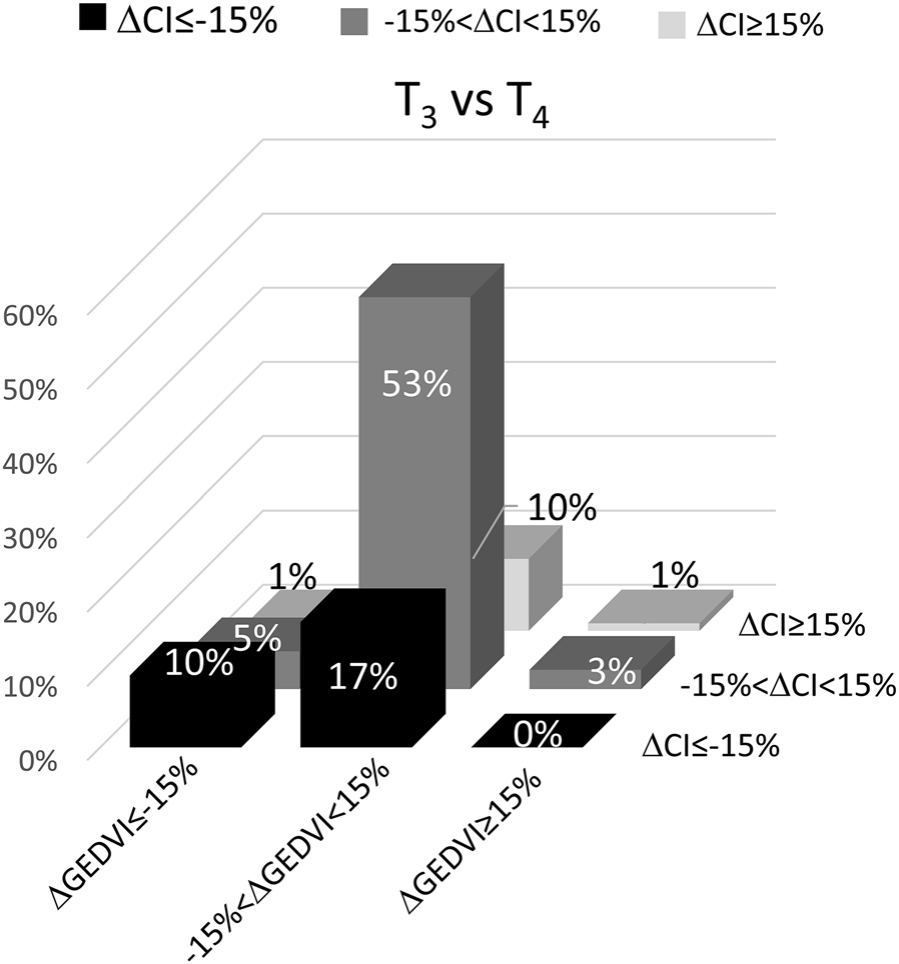
Hemodynamic pattern as a function of ΔCI and GEDVI between T_3_ and T_4_. Bars are percentage of patients falling in each category. CI, cardiac index; GEDVI, global end-diastolic volume index; ΔCI, change in CI; ΔGEDVI, change in GEDVI; T_3_, end of prone position session; T_4_, after prone position session

CI decreased between T_3_ and T_4_ in 27% of the sessions and was associated with a decrease in GEDVI in 10% of the sessions and with a stable GEDVI in 17% of the sessions (Fig. 4).

Change in CI between T_1_ and T_3_ was not correlated with change in CI between T_3_ and T_4_, while change in GEDVI between T_1_ and T_3_ was negatively correlated with change in GEDVI between T_3_ and T_4_ (Additional file 7: Figure S3).

## Discussion

The main findings of the study are the following: (1) Early and late hemodynamic response to PP are strongly related; (2) CI significantly increases at the end of 25% of PP sessions; (3) PP is associated with a slight yet sustained increase in GEDVI, reversible after return in SP, unrelated to fluid administration; (4) return to SP is associated with a small but significant decrease in CI and oxygen delivery; (5) PP may improve cardiovascular status by increasing cardiac preload and hence CI in patients with preload responsiveness.

### Effect of prone position on CI

The study results are in line with previous smaller studies showing the lack of significant impact of the shift from SP to PP on CI in unselected ARDS patients [4–10]. Still, 25% of the PP sessions were associated with a significant increase in CI at T_3_ compared to baseline, of which approximately half were related to an increase in GEDVI (and presumably an increase in cardiac preload). Of note, most of these patients increased their GEDVI without receiving any fluid challenge. The remaining half displayed no detectable change in GEDVI, suggesting an improvement in CI unrelated to cardiac preload. It may be speculated that this latter group of patients might encompass patients with various degrees of pulmonary vascular system dysfunction, benefiting from the unloading of their right ventricle in response to PP [12]. Of note, the rate of PP sessions associated with an increase in CI related to cardiac preload increase at T_3_ is substantially lower in the present study than in Jozwiak et al. [13]. Besides differences in case mix, timing of measurements, and PEEP management, a difference in the starting position (strict supine in the present study vs. 45° semi-recumbent) may partly explain this discrepancy. Oppositely, 23% of the PP sessions were associated with a significant decrease in CI at T_3_ compared to baseline, the majority of which without detectable change in GEDVI. The significantly greater CI, GEDVI, and vasopressor dose at baseline in this subgroup of patients suggest that the decrease in CI during PP may be related to resolution of an hyperdynamic state during the PP session. Finally, the high concordance rate between early and late cardiac index changes suggests that hemodynamic response to PP is persistent throughout the whole PP session.

Unlike previous smaller studies [4, 6, 11], the present study identified a small but significant decrease in CI and oxygen delivery during the shift from PP to SP. Apart from differences in case mix, ventilatory settings, or timing of measurement, it is likely that previous studies were strongly underpowered to detect this effect. Our data suggest that this decrease is related to a decrease in preload in approximately 1/3 of the sessions, suggesting reversal of the PP-related preload improvement effect identified by Jozwiak et al. [13].

### Effect of prone position on GEDVI

We observed a slight but significant increase in GEDVI at the end of the PP session, reversible after return to the SP, in line with a previous smaller study [33]. Since PP modifies regional ventilation–perfusion ratios [34], it has been speculated that this slight increase in GEDVI might be related to an increase in pulmonary thermal volume in PP. The lack of significant difference regarding this parameter between T_1_, T_2_ and T_3_ does not support this hypothesis, along with the fact that most of the PP sessions with significant increase in GEDVI between T_1_ and T_3_ were associated with an increase in CI. This suggests that changes in GEDVI during the PP session remain a reliable indicator of changes in preload.

It might be questioned whether the slight increase in GEDVI related to the shift from supine to PP is associated with a meaningful increase in cardiac preload. Since this increase is of similar magnitude than that provided by a 500-mL fluid challenge [35, 36], it suggests that the observed change in cardiac preload related to PP is clinically relevant. Furthermore, our study confirms the positive effect of PP on cardiac preload previously shown in a smaller study within 20 min after the postural change [13] and extends this finding up to the end of longer PP sessions [1].

### Strengths and limits

Some limitations of the present study should be acknowledged. First, the retrospective feature of the study explains the heterogeneity between patients regarding assessment time points and the high rate of missing values for some variables. Second, the present study selected a subpopulation of ARDS patients with acute circulatory failure requiring PiCCO^®^ monitoring, making a selection bias uncontrolled for. Third, the observational design does not allow to control for the effect of time. Fourth, some important variables are lacking (pulmonary artery pressure, comprehensive evaluation of right ventricle function, assessment of preload reserve status, etc.), hindering interpretation of hemodynamic data. Five, co-interventions (such as fluid loading, change in vasopressor dose) during study could have interfered with PP effect on hemodynamics, although these confounders were accounted for in the statistical analysis. Sixth, a 15% conservative threshold was used to detect significant changes in CI and GEDVI, since the least significant change detectable by thermodilution is around 12% when three boluses are used for PiCCO^®^ calibration [19], thereby limiting the potential of this technique for detecting hemodynamic changes. Seven, the rate of patients with ACP was low in the present study (9%), as compared to 22% in a recent multicenter study [14], and may be partly explained by the lack of systematic daily ultrasound evaluation in the present study, but may be also related to higher tidal volumes (6.2 vs. 6.8 mL kg^−1^) and driving pressures (12 vs. 16 cm H_2_O) in the latter, in addition to a lower use of PP (100% vs. 29%).

Nevertheless, the number of studied PP sessions outranks by almost one order of magnitude previous studies on the effect of PP on CI. This substantial size allowed to compute rates of CI response to PP with relatively narrow confidence intervals and to perform multivariate analysis of factors associated with CI variations, allowing control of confounding variables.

### Clinical implications

The present study identified a beneficial effect of PP on CI in 25% of the sessions, especially in patients with lower CI before PP, associated with an increase in cardiac preload. This suggests that hemodynamic instability should not be an obstacle to PP. Since return to SP may be associated with a decrease in CI in approximately a quarter of the PP sessions, serial evaluations of CI and fluid responsiveness may be recommended during this period.

## Conclusions

Prone position is associated with an increase in CI in 18% to 32% of the PP sessions and a sustained increase in GEDVI, both reversible after return to SP. PP may improve CI by increasing cardiac preload in patients with preload responsiveness. Return from PP to SP is associated with a slight hemodynamic impairment, at least partly related to decreased cardiac preload.

## Additional files

**Additional file 1.** Missing values per variable.

**Additional file 2.** Univariate analysis of cardiac index response to prone position.

**Additional file 3.** Diagnostic performance of variables assessed at T_1_ to predict an increase in CI greater than 15% between T_1_ and T_3_.

**Additional file 4.** Diagnostic performance of variables assessed at T_1_ to predict a decrease in CI greater than 15% between T_1_ and T_3_.

**Additional file 5.** Cardiac index response to prone position in the 40 patients with multiple prone position sessions.

**Additional file 6.** Correlation between CI and GEDVI changes between T_1_ and T_3_.

**Additional file 7.** Change in cardiac index and global end-diastolic volume between T_1_ and T_3_ and between T_3_ and T_4_.

**Additional file 8.** Dataset.

## Abbreviations

ACP: acute cor pulmonale
ARDS: acute respiratory distress syndrome
AUC: area under ROC curve
CI: cardiac index
CI_95%_: 95% confidence interval
GEDVI: global end-diastolic volume index
ICU: intensive care unit
PEEP: positive end-expiratory pressure
PP: prone position
SP: supine position
