# Supplementary material for: Hemodynamic effects of extended prone position sessions in ARDS
Source: Ann Intensive Care. 2018 Dec 7;8:120. doi: 10.1186/s13613-018-0464-9 (PMC6286298; doi:10.1186/s13613-018-0464-9)
Supplement: Supplementary file 1 — Additional file 1. Missing values per variable. [file 13613_2018_464_MOESM1_ESM.docx]

**Additional file 1: Table S1. Missing values per variable.**

| Variables | Count (%) |
| --- | --- |
| Age | 0 (0%) |
| Sex | 0 (0%) |
| Body weight at ICU admission  Body weight at PPS onset  Cumulative fluid balance at PPS onset  Fluid balance during PPS | 0 (0%)  17 (9%)  7 (9%)  45 (23%) |
| Height | 0 (0%) |
| SAPSII score | 0 (0%) |
| Duration of PPS  SOFA score the day of PPS | 0 (0%)  0 (0%) |
| Adjunctive therapies during PPS | 0 (0%) |
| Acute cor pulmonale | 0 (0%) |
| Heart rate at T_1_ | 0 (0%) |
| Heart rate at T_2_ | 0 (0%) |
| Heart rate at T_3_ | 0 (0%) |
| Heart rate at T_4_ | 0 (0%) |
| Mean arterial pressure at T_1_ | 0 (0%) |
| Mean arterial pressure at T_2_ | 0 (0%) |
| Mean arterial pressure at T_3_ | 0 (0%) |
| Mean arterial pressure at T_4_ | 0 (0%) |
| Central venous pressure at T_1_ | 32 (16%) |
| Central venous pressure at T_2_ | 23 (12%) |
| Central venous pressure at T_3_ | 21 (11%) |
| Central venous pressure at T_4_ | 19 (10%) |
| Cardiac index at T_1_ | 0 (0% |
| Cardiac index at T_2_ | 3 (2%) |
| Cardiac index at T_3_ | 0 (0%) |
| Cardiac index at T_4_ | 0 (0%) |
| Global ejection fraction at T_1_ | 0 (0%) |
| Global ejection fraction at T_2_ | 2 (1%) |
| Global ejection fraction at T_3_ | 2 (1%) |
| Global ejection fraction at T_4_ | 0 (0%) |
| Global end diastolic volume index at T_1_ | 0 (0%) |
| Global end diastolic volume index at T_2_ | 2 (1%) |
| Global end diastolic volume index at T_3_ | 2 (1%) |
| Global end diastolic volume index at T_4_ | 0 (0%) |
| Cardiac function index at T_1_ | 0 (0%) |
| Cardiac function index at T_2_ | 3 (2%) |
| Cardiac function index at T_3_ | 2 (1%) |
| Cardiac function index at T_4_ | 0 (0%) |
| Pulmonary vascular permeability index at T_1_ | 0 (0%) |
| Pulmonary vascular permeability index at T_2_ | 2 (1%) |
| Pulmonary vascular permeability index at T_3_ | 2 (1%) |
| Pulmonary vascular permeability index at T_4_ | 0 (0%) |
| Pulmonary thermal volume at T_1_ | 0 (0%) |
| Pulmonary thermal volume at T_2_ | 2 (1%) |
| Pulmonary thermal volume at T_3_ | 2 (1%) |
| Pulmonary thermal volume at T_4_ | 0 (0%) |
| Intrathoracic thermal volume at T_1_ | 0 (0%) |
| Intrathoracic thermal volume at T_2_ | 2 (1%) |
| Intrathoracic thermal volume at T_3_ | 2 (1%) |
| Intrathoracic thermal volume at T_4_ | 0 (0%) |
| Extra vascular lung water index at T_1_ | 0 (0%) |
| Extra vascular lung water index at T_2_ | 2 (1%) |
| Extra vascular lung water index at T_3_ | 0 (0%) |
| Extra vascular lung water index at T_4_ | 0 (0%) |
| Vasopressor dose at T_1_ | 0 (0%) |
| Vasopressor dose at T_2_ | 3 (2%) |
| Vasopressor dose at T_3_ | 0 (0%) |
| Vasopressor dose at T_4_ | 2 (1%) |
| Dobutamine dose at T_1_ | 0 (0%) |
| Dobutamine dose at T_2_ | 1 (1%) |
| Dobutamine dose at T_3_ | 0 (0%) |
| Dobutamine dose at T_4_ | 0 (0%) |
| Fluid challenge volume at T_1_ | NA |
| Dobutamine dose at T_2_ | 0 (0%) |
| Dobutamine dose at T_3_ | 0 (0%) |
| Dobutamine dose at T_4_ | 0 (0%) |
| Tidal volume at T_1_ | 0 (0%) |
| Tidal volume at T_3_ | 0 (0%) |
| Tidal volume at T_4_ | 0 (0%) |
| Respiratory rate at T_1_ | 11 (6%) |
| Respiratory rate at T_3_ | 12 (6%) |
| Respiratory rate at T_4_ | 12 (6%) |
| I:E ratio at T_1_ | 37 (19%) |
| I:E ratio at T_3_ | 37 (19%) |
| I:E ratio at T_4_ | 36 (18%) |
| PEEP at T_1_ | 0 (0%) |
| PEEP at T_3_ | 0 (0%) |
| PEEP at T_4_ | 0 (0%) |
| Plateau pressure at T_1_ | 56 (28%) |
| Plateau pressure at T_3_ | 45 (23%) |
| Plateau pressure at T_4_ | 69 (35%) |
| Total PEEP of the respiratory system at T_1_ | 113 (57%) |
| Total PEEP of the respiratory system at T_3_ | 92 (47%) |
| Total PEEP of the respiratory system at T_4_ | 120 (61%) |
| Driving pressure at T_1_ | 113 (57%) |
| Driving pressure at T_3_ | 92 (47%) |
| Driving pressure at T_4_ | 120 (61%) |
| pH at T_1_ | 0 (0%) |
| pH at T_3_ | 0 (0%) |
| pH at T_4_ | 4 (2%) |
| PaO_2_ at T_1_ | 0 (0%) |
| PaO_2_ at T_3_ | 0 (0%) |
| PaO_2_ at T_4_ | 4 (2%) |
| PaO_2_/FiO_2_ at T_1_ | 0 (0%) |
| PaO_2_/FiO_2_ at T_3_ | 0 (0%) |
| PaO_2_/FiO_2_ at T_4_ | 4 (2%) |
| PaCO_2_ at T_1_ | 0 (0%) |
| PaCO_2_ at T_3_ | 0 (0%) |
| PaCO_2_ at T_4_ | 4 (2%) |
| Lactate at T_1_ | 10 (5%) |
| Lactate at T_3_ | 15 (8%) |
| Lactate at T_4_ | 21 (11%) |
| Arterial oxygen transport at T_1_ | 0 (0%) |
| Arterial oxygen transport at T_3_ | 0 (0%) |
| Arterial oxygen transport at T_4_ | 4 (2%) |

ARDS: acute respiratory distress syndrome; PEEP: positive end-expiratory pressure; PPS: prone position session; SAPSII: simplified acute physiology score II; SOFA: sepsis-related organ failure assessment; T_1_: before prone position session, T_2_: at the beginning of prone position session; T_3_: at the end of prone position session; T_4_: after return to supine position.
