# Supplementary material for: Hemodynamic effects of extended prone position sessions in ARDS
Source: Ann Intensive Care. 2018 Dec 7;8:120. doi: 10.1186/s13613-018-0464-9 (PMC6286298; doi:10.1186/s13613-018-0464-9)
Supplement: Supplementary file 2 — Additional file 2. Univariate analysis of cardiac index response to prone position. [file 13613_2018_464_MOESM2_ESM.docx]

**Additional file 2: Table S2.** **Univariate analysis of cardiac index response to prone position.**

| Variables | Decrease in CI ≥ 15% between T_1_ and T_3_  n=46 (23%) | No significant change in cardiac index between T_1_ and T_3_  n=102 (52%) | Increase in CI ≥ 15% between T_1_ and T_3_  n=49 (25%) | p value |
| --- | --- | --- | --- | --- |
| Age (year) | 64 ± 13 | 64 ± 11 | 65 ± 12 | 0.88 |
| Male sex | 38 (83%) | 71 (70%) | 26 (53%) | 0.90 |
| BMI (kg.m^-2^) | 30 ± 7 | 31 ± 7 | 30 ± 7 | 0.87 |
| SAPS II | 62 ± 16 | 59 ± 14 | 64 ± 18 | 1 |
| Admission category |  |  |  | 1 |
| - medical | 45 (98%) | 100 (98%) | 47 (96%) |  |
| - other | 1 (2%) | 2 (2%) | 2 (4%) |  |
| ARDS severity |  |  |  | 0.95 |
| - severe | 35 (76%) | 87 (85%) | 36 (74%) |  |
| - moderate | 11 (24%) | 15 (15%) | 13 (27%) |  |
| Pneumonia as ARDS risk factor | 34 (74%) | 75 (74%) | 36 (73%) | 1 |
| Aspiration as ARDS risk factor | 11 (24%) | 34 (33%) | 19 (39%) | 1 |
| Non pulmonary sepsis as ARDS risk factor | 4 (9%) | 11 (11%) | 7 (14%) | 1 |
| Time between ARDS onset and PP session onset (day) | 2 ± 3 | 3 ± 3 | 3 ± 3 | 0.17 |
| PP session duration (hour) | 16 ± 3 | 16 ± 2 | 16 ± 2 | 0.76 |
| SOFA score | 15 ± 3 | 14 ± 4 | 14 ± 4 | 0.11 |
| Cumulative fluid balance at PP session onset (kg) | 3.3 ± 6.2 | 2.9 ± 6.9 | 0 ± 5.5 | 0.16 |
| Fluid balance during PP session (kg) | 1.7 ± 4.1 | 0.5 ± 3.0 | 0.7 ± 3.2 | 0.19 |
| Renal replacement therapy | 23 (50%) | 41 (40%) | 20 (41%) | 0.34 |
| Dobutamine administration | 17 (37%) | 29 (28%) | 16 (33%) | 0.45 |
| Vasopressor administration | 43 (93%) | 83 (81%) | 40 (82%) | 0.14 |
| Inhaled nitric oxide | 11 (24%) | 18 (18%) | 10 (20%) | 0.49 |
| Neuromuscular blocking agent | 42 (91%) | 93 (91%) | 47 (96%) | 0.28 |
| Volume of fluid challenges between T_1_ and T_3_ (mL) | 322 ± 533 | 251 ± 754 ‡ | 708 ± 1363 † | **< 0.01** |
| HR at T_1_ (min^-1^) | 105 ± 23 | 98 ± 22 | 95 ± 23 | 0.10 |
| MAP at T_1_ (mm Hg) | 75 ± 9 | 76 ± 11 | 77 ± 10 | 0.62 |
| CVP at T_1_ (mm Hg) | 13 ± 5 | 13 ± 5 | 14 ± 5 | 0.79 |
| CI at T_1_ (L.min^-1^.m^-2^) | 4.1 ± 1.4 | 3.6 ± 1.2 †,‡ | 2.6 ± 0.7 † | **<0.001** |
| EVLWI at T_1_ (mL.kg^-1^ PBW) | 15.0 ± 4.5 | 13.2 ± 4.1 | 13.8 ± 4.7 | 0.23 |
| PVPI at T_1_ | 2.6 ± 0.9 | 2.5 ± 0.9 | 2.8 ± 1.4 | 0.39 |
| GEDVI at T_1_ (mL.m^-2^) | 805 ± 252 | 704 ± 170 †,‡ | 672 ± 150 † | **<0.001** |
| CFI at T_1_ (min^-1^) | 5.4 ± 1.9 | 5.3 ± 1.9 †,‡ | 4.1 ± 1.4 † | **<0.001** |
| GEF at T_1_ (%) | 21 ± 8 | 22 ± 7 ‡ | 19 ± 7 † | **<0.05** |
| PTV at T_1_ (mL) | 1304 ± 363 | 1111 ± 331 | 1138 ± 396 | 0.05 |
| ITTV at T_1_ (mL) | 2836 ± 723 | 2466 ± 622 † | 2413 ± 569 † | **<0.001** |
| Vasopressor dose at T_1_ (µg.kg^-1^.min^-1^) | 1.29 ± 1.84 | 0.69 ± 1.09 † | 1.04 ± 2.31 † | **< 0.01** |
| Dobutamine dose at T_1_ (µg.kg^-1^.min^-1^) | 2.37 ± 5.22 | 1.86 ± 4.48 | 4.39 ± 8.61 | 0.19 |
| Lactate at T_1_ (mmol.L^-1^) | 4.2 ± 3.9 | 3.2 ± 2.6 | 3.7 ± 3.7 | 0.69 |
| pH at T_1_ | 7.33 ± 0.11 | 7.36 ± 0.09 | 7.34 ± 0.10 | 0.14 |
| PaCO_2_ at T_1_ (mm Hg) | 45 ± 13 | 45 ± 9 | 44 ± 10 | 0.29 |
| PaO_2_/FiO_2_ at T_1_ (mm Hg) | 104 ± 28 | 113 ± 26 | 116 ± 32 | 0.21 |
| O_2_ responders to PP | 36 (78%) | 83 (81%) | 38 (78%) | 0.84 |
| CO_2_ responders to PP | 23 (50%) | 53 (52%) | 21 (43%) | 0.56 |
| O_2_ and CO_2_ responders to PP | 20 (43%) | 45 (44%) | 20 (41%) | 0.97 |
| DO_2_ at T_1_ (mL.min.m^-2^) | 479 ± 156 | 428 ± 143 †,‡ | 334 ± 96 † | **<0.001** |
| VT at T_1_ (mL.kg^-1^ PBW) | 6.1 ± 0.8 | 6.1 ± 0.7 | 6.2 ± 0.7 | 0.39 |
| PEEP at T_1_ (cm H_2_O) | 9 ± 4 | 10 ± 2 | 10 ± 3 | 0.65 |
| PEEPtot at T_1_ (cm H_2_O) | 10 ± 3 | 11 ± 2 | 11 ± 2 | 0.32 |
| Pplat at T_1_ (cm H_2_O) | 24 ± 5 | 23 ± 4 | 22 ± 4 | 0.34 |
| ΔP at T_1_ (cm H_2_O) | 12 ± 4 | 11 ± 3 | 11 ± 4 | 0.36 |

† p < 0.05 vs. decrease in CI; ‡ p < 0.05 vs. increase in CI.

ARDS = acute respiratory distress syndrome; BMI = body mass index; CFI = cardiac function index; CI = cardiac index; CVP = central venous pressure; DO_2_ = oxygen delivery; ΔP = driving pressure; EVLWI = extravascular lung water index; FiO_2_ = inspired oxygen fraction; GEDVI = global end-diastolic volume index; GEF = global ejection fraction; HR = heart rate; ITTV = intrathoracic thermal volume; MAP = mean arterial pressure; PaCO_2_ = partial pressure of arterial carbon dioxide; PaO_2_ = partial pressure of arterial oxygen; PBW = predicted body weight; PEEP = positive end-expiratory pressure; PEEPtot= total PEEP of the respiratory system; PP = prone position; Pplat = plateau pressure of the respiratory system; PTV = pulmonary thermal volume; PVPI = pulmonary vascular permeability index; SAPSII = simplified acute physiology score II; SOFA = sepsis-related organ failure assessment; T_1_ = before prone position, T_2_ = beginning of prone position session; T_3_ = end of prone position session; T_4_ = after prone position session; VT = tidal volume.
