# Supplementary material for: Hemodynamic effects of extended prone position sessions in ARDS
Source: Ann Intensive Care. 2018 Dec 7;8:120. doi: 10.1186/s13613-018-0464-9 (PMC6286298; doi:10.1186/s13613-018-0464-9)
Supplement: Supplementary file 3 — Additional file 3. Diagnostic performance of variables assessed at T1 to predict an increase in CI greater than 15% between T1 and T3. [file 13613_2018_464_MOESM3_ESM.docx]

**Additional file 3: Table S3.** **Diagnostic performance of variables assessed at T_1_ to predict an increase in CI greater than 15% between T_1_ and T_3_.**

|  | AUC  [CI_95%_] | Best threshold | Se  [CI_95%_] | Sp  [CI_95%_] | PLR  [CI_95%_] | NLR  [CI_95%_] |
| --- | --- | --- | --- | --- | --- | --- |
| CI at T_1_  (mL.min^-1^.m^-2^) | 0.79  [0.73-0.86] | 2.8 | 0.69  [0.55-0.82] | 0.76  [0.69-0.83 | 2.9  [2.1-4.1] | 0.4  [0.26-0.61] |
| GEDVI at T_1_ (mL.m^-2^) | 0.58  [0.49-0.67] | 725 | 0.71  [0.57-0.83] | 0.43  [0.35-0.51] | 1.26  [1.0-1.6] | 0.7  [0.4-1.1] |
| CFI at T_1_  (min^-1^) | 0.71  [0.63-0.79] | 5.0 | 0.88  [0.75-0.95] | 0.50  [0.42-0.58] | 1.8  [1.5-2.1] | 0.2  [0.1-0.5] |
| GEF at T_1_  (%) | 0.61  [0.51-0.70] | 20 | 0.59  [0.50-0.66] | 0.58  [0.50-0.66] | 1.4  [1.0-1.9] | 0.7  [0.5-1.0] |
| IITV at T_1_ (mL) | 0.56  [0.47-0.66] | 3125 | 0.92  [0.80-0.98] | 0.22  [0.16-0.30] | 1.2  [1.0-1.3] | 0.4  [0.1-1.0] |
| Vasopressor dose at T_1_ (µg.kg^-1^.min^-1^) | 0.58  [0.48-0.67] | 0.16 | 0.51  [0.36-0.66] | 0.72  [0.64-0.79] | 1.8  [1.3-2.7] | 0.7  [0.5-0.9] |
| DO_2_ at T_1_  (mL.min.m^-2^) | 0.75  [0.67-0.82] | 382 | 0.84  [0.70-0.93] | 0.64  [0.57-0.73] | 2.4  [1.9-3.1] | 0.3  [0.1-0.5] |

AUC = area under ROC curve; CFI = cardiac function index; CI = cardiac index; CI_95%_ = 95% confidence interval; DO_2_ = oxygen delivery; GEDVI = global end-diastolic volume index; GEF = global ejection fraction; ITTV = intrathoracic thermal volume; NLR = negative likelihood ratio; PLR = positive likelihood ratio; Se = sensitivity; Sp = specificity; T_1_ = before prone position; T_3_ = end of prone position session.
