# Supplementary material for: Hemodynamic effects of extended prone position sessions in ARDS
Source: Ann Intensive Care. 2018 Dec 7;8:120. doi: 10.1186/s13613-018-0464-9 (PMC6286298; doi:10.1186/s13613-018-0464-9)
Supplement: Supplementary file 4 — Additional file 4. Diagnostic performance of variables assessed at T1 to predict a decrease in CI greater than 15% between T1 and T3. [file 13613_2018_464_MOESM4_ESM.docx]

**Additional file 4: Table S4.** **Diagnostic performance of variables assessed at T_1_ to predict a decrease in CI greater than 15% between T_1_ and T_3_.**

|  | AUC  [CI_95%_] | Best threshold | Se  [CI_95%_] | Sp  [CI_95%_] | PLR  [CI_95%_] | NLR  [CI_95%_] |
| --- | --- | --- | --- | --- | --- | --- |
| CI at T_1_  (mL.min^-1^.m^-2^) | 0.68  [0.59-0.77] | 3.5 | 0.63  [0.48-0.77] | 0.70  [0.62-0.77] | 2.11  [1.5-2.9] | 0.5  [0.4-0.8] |
| GEDVI at T_1_ (mL.m^-2^) | 0.64  [0.55-0.73] | 679 | 0.70  [0.54-0.82] | 0.55  [0.47-0.63] | 1.5  [1.2-2.0] | 0.6  [0.3-0.9] |
| CFI at T_1_  (min^-1^) | 0.58  [0.48-0.68] | 5.4 | 0.46  [0.31-0.60] | 0.72  [0.63-0.79] | 1.6  [1.1-2.4] | 0.8  [0.7-0.9] |
| GEF at T_1_  (%) | 0.52  [0.42-0.62] | 24 | 0.76  [0.61-0.87] | 0.33  [0.26-0.41] | 1.1  [0.9-1.4] | 0.7  [0.4-1.3] |
| IITV at T_1_ (mL) | 0.67  [0.58-0.75] | 2416 | 0.78  [0.64-0.89] | 0.60  [0.51-0.68] | 1.9  [1.5-2.5] | 0.4  [0.2-0.6] |
| Vasopressor dose at T_1_ (µg.kg^-1^.min^-1^) | 0.61  [0.51-0.70] | 0.49 | 0.59  [0.43-0.73] | 0.63  [0.55-0.71] | 1.6  [1.2-2.2] | 0.7  [0.5-0.9] |
| DO_2_ at T_1_  (mL.min.m^-2^) | 0.68  [0.59-0.77] | 352 | 0.85  [0.71-0.94] | 0.46  [0.38-0.54] | 1.6  [1.3-1.9] | 0.3  [0.2-0.7] |

AUC = area under ROC curve; CFI = cardiac function index; CI = cardiac index; CI_95%_ = 95% confidence interval; DO_2_ = oxygen delivery; GEDVI = global end-diastolic volume index; GEF = global ejection fraction; ITTV = intrathoracic thermal volume; NLR = negative likelihood ratio; PLR = positive likelihood ratio; Se = sensitivity; Sp = specificity; T_1_ = before prone position; T_3_ = end of prone position session.
