# Supplementary material for: Hemodynamic effects of extended prone position sessions in ARDS
Source: Ann Intensive Care. 2018 Dec 7;8:120. doi: 10.1186/s13613-018-0464-9 (PMC6286298; doi:10.1186/s13613-018-0464-9)
Supplement: Supplementary file 5 — Additional file 5. Cardiac index response to prone position in the 40 patients with multiple prone position sessions. [file 13613_2018_464_MOESM5_ESM.docx]

**Additional file 5: Figure S1.** **Cardiac index response to prone position in the 40 patients with multiple prone position sessions.**


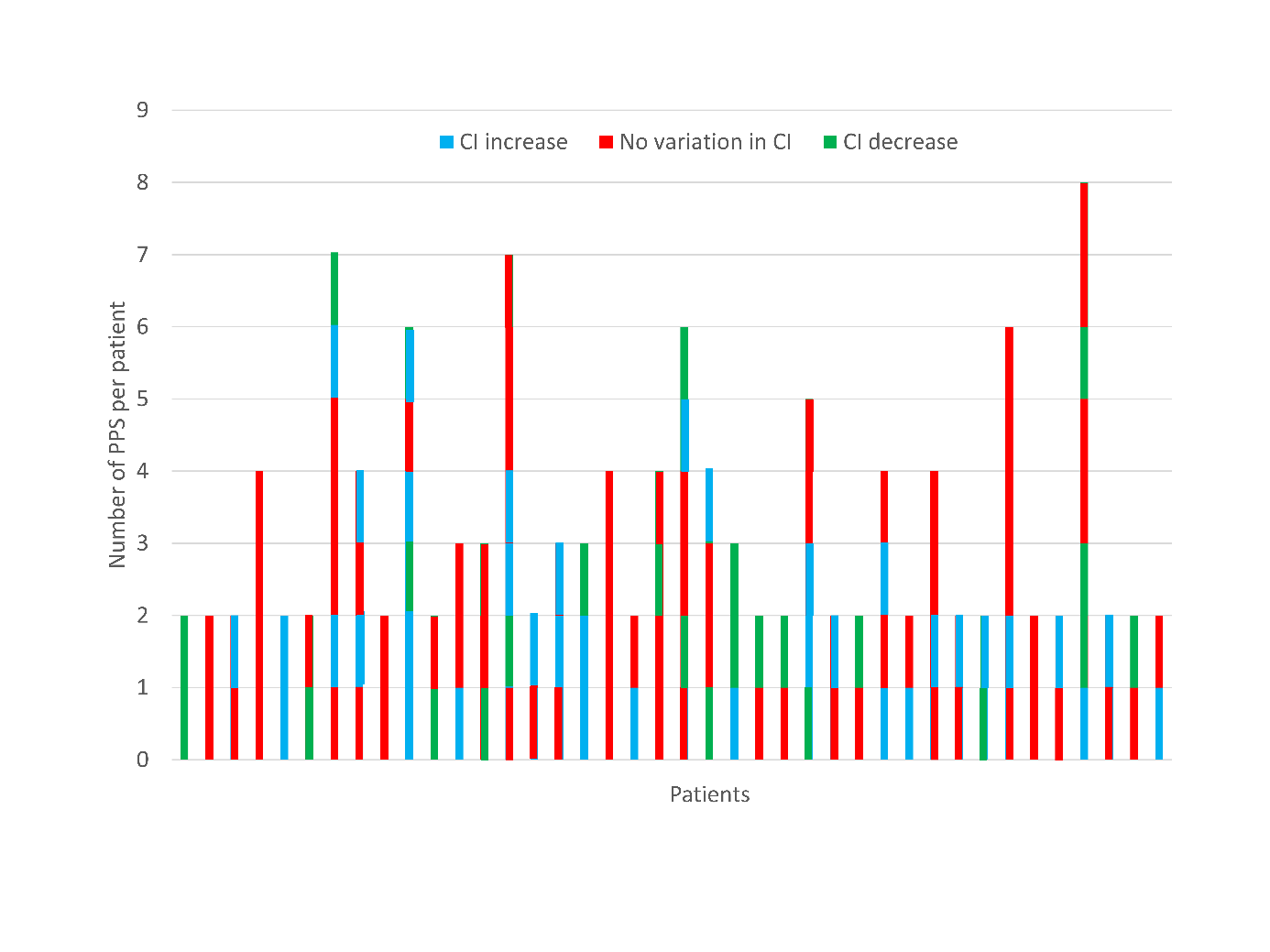


Each bar refers to one patient.

CI = cardiac index; PPS = prone position session.
