# Supplementary material for: Hemodynamic effects of extended prone position sessions in ARDS
Source: Ann Intensive Care. 2018 Dec 7;8:120. doi: 10.1186/s13613-018-0464-9 (PMC6286298; doi:10.1186/s13613-018-0464-9)
Supplement: Supplementary file 6 — Additional file 6. Correlation between CI and GEDVI changes between T1 and T3. [file 13613_2018_464_MOESM6_ESM.docx]

**Additional file 6: Figure S2. Correlation between CI and GEDVI changes between T_1_ and T_3_.**


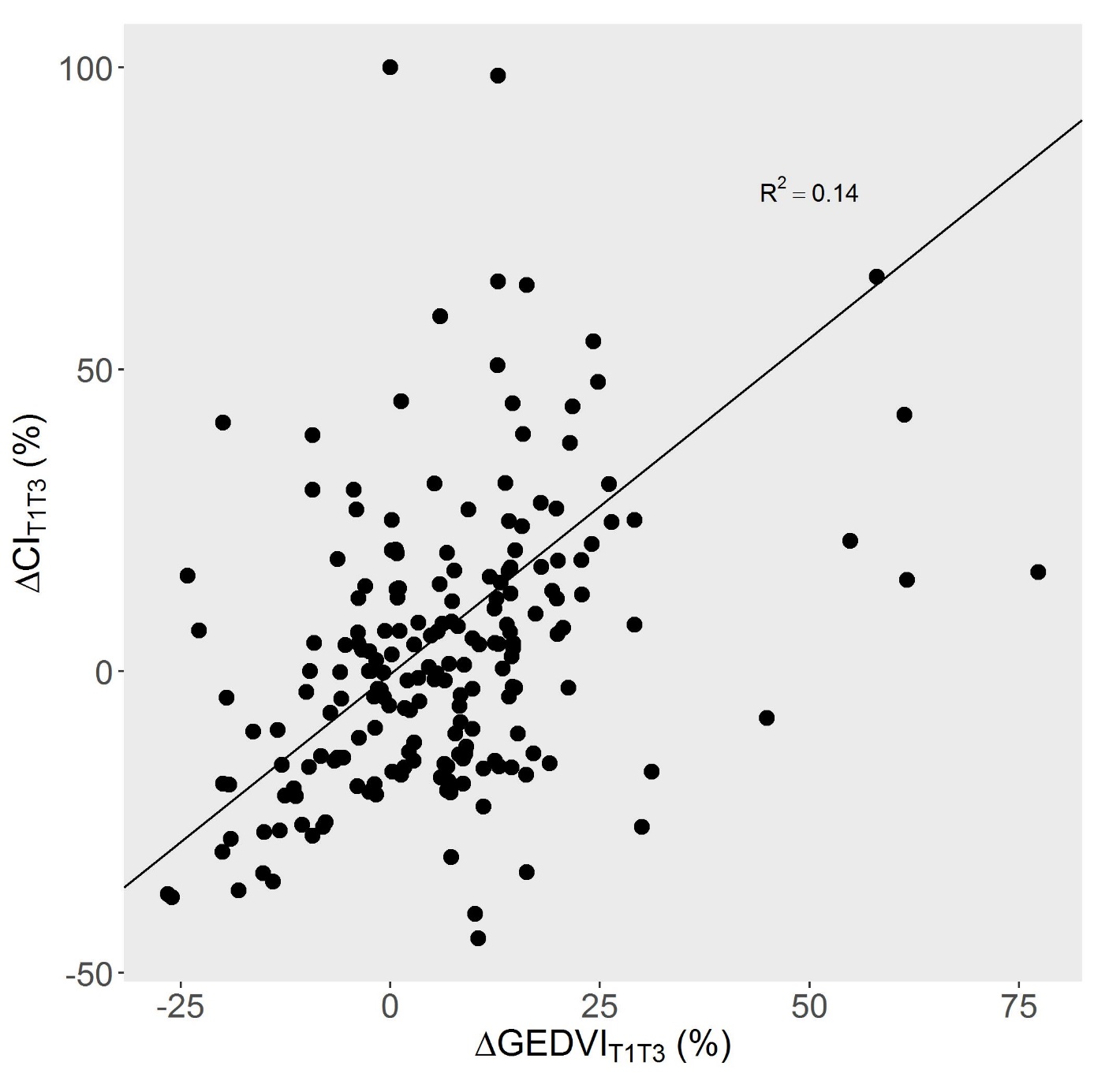


Dot are individual datapoints. Line is regression line with corresponding R^2^. Correlation is statistically significant with p values < 0.001.

ΔCI_T1T3_ = change in cardiac index between T_1_ and T_3_; ΔGEDVI_T1T3_ = change in global end-diastolic volume index between T_1_ and T_3_.

**Supplementary figure 3. Correlation between CI and GEDVI changes between T_1_ and T_3_ as a function of changes in cardiac function index.**


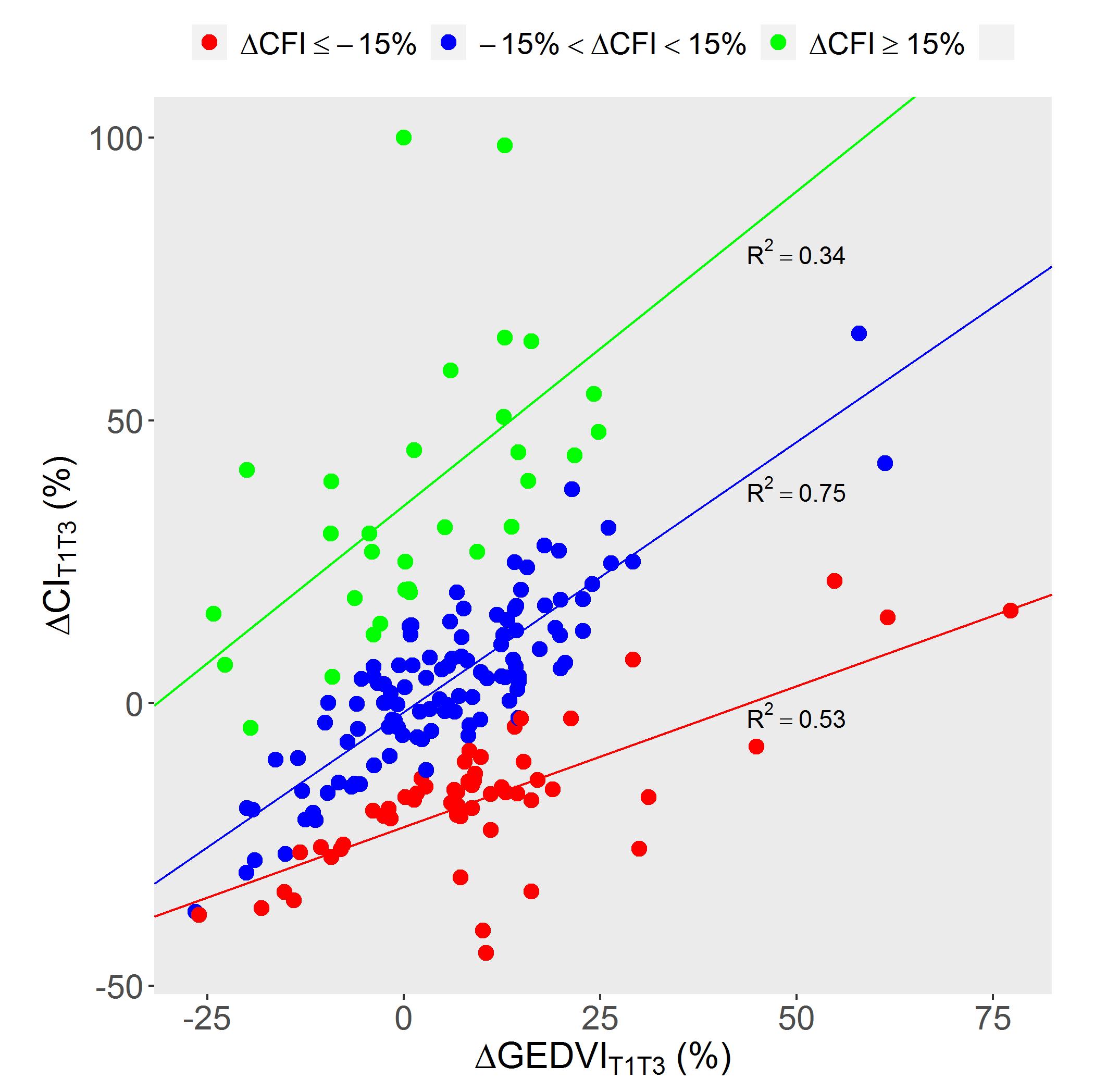


Dot are individual datapoints. Green dots refer to sessions with significant increase in CFI (≥15%) between T_1_ and T_3_ suggesting a change in CI not fully related to GEDVI change. Blue dots refer to sessions without significant increase in CFI between T1 and T3. Red dots refer to sessions with significant decrease in CFI (≤-15%) between T1 and T3. Lines are regression lines in each subgroup with corresponding R^2^. All correlations are statistically significant with p values < 0.001.

ΔCFI = change in cardiac function index between T_1_ and T_3_; ΔCI_T1T3_ = change in cardiac index between T_1_ and T_3_; ΔGEDVI_T1T3_ = change in global end-diastolic volume index between T_1_ and T_3_
