# Supplementary material for: Hemodynamic effects of extended prone position sessions in ARDS
Source: Ann Intensive Care. 2018 Dec 7;8:120. doi: 10.1186/s13613-018-0464-9 (PMC6286298; doi:10.1186/s13613-018-0464-9)
Supplement: Supplementary file 7 — Additional file 7. Change in cardiac index and global end-diastolic volume between T1 and T3 and between T3 and T4. [file 13613_2018_464_MOESM7_ESM.docx]

**Additional file 7: Figure S3.** **Change in cardiac index and global end-diastolic volume between T_1_ and T_3_ and between T_3_ and T_4_.**


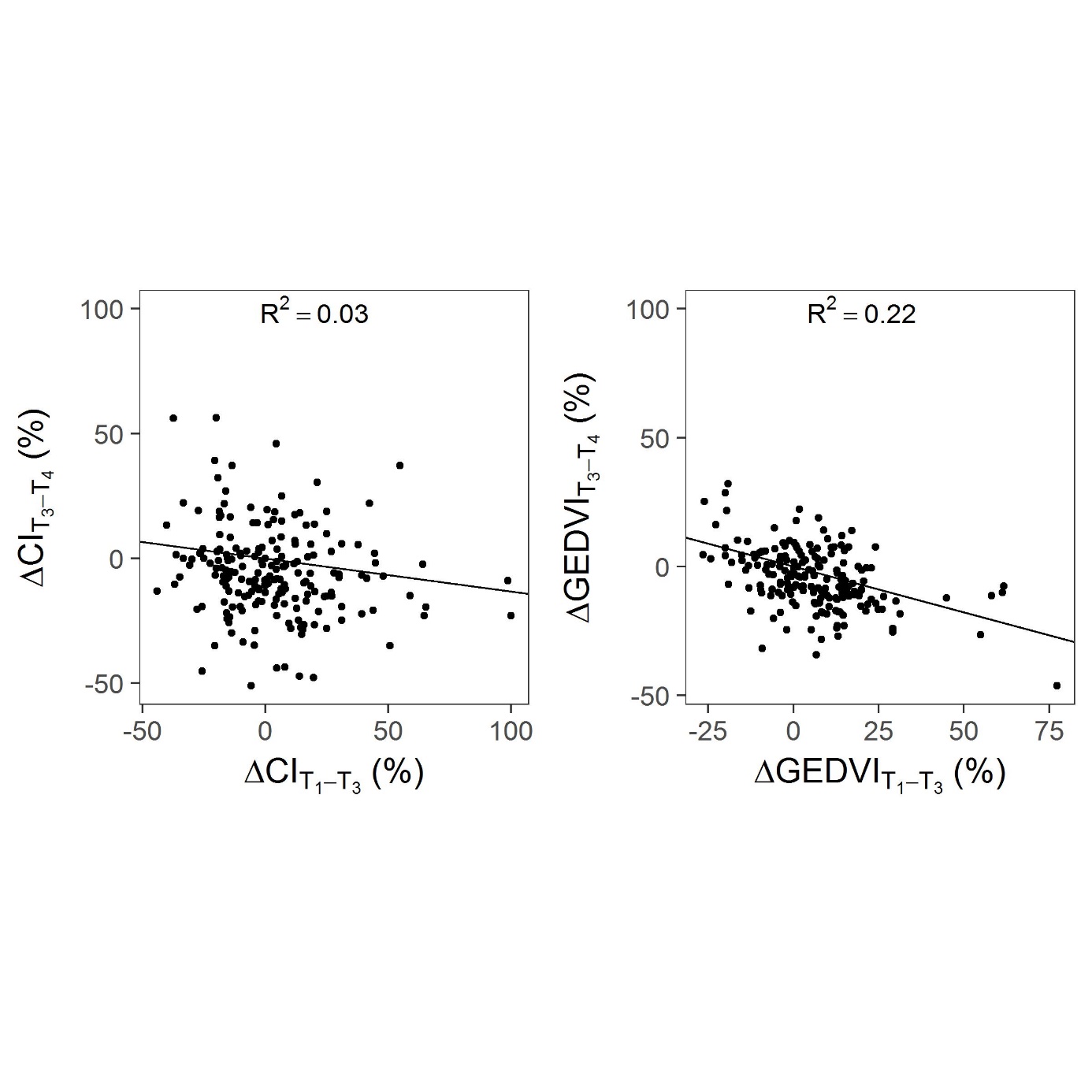


Symbols are individual values for each prone position session. Solid lines are regression lines.

ΔCI_T1-T3_ = change in cardiac index between T_1_ and T_3_, ΔCI_T3-T4_= change in cardiac index between T_3_ and T_4_, ΔGEDVI_T1-T3_= change in global-end diastolic volume between T_1_ and T_3_, ΔGEDVI_T3-T4_= change in global-end diastolic volume between T_3_ and T_4_; R^2^ = coefficient of determination; T_1_ = before prone position; T_3_ = end of prone position session; T_4_ = after prone position session.
